# Fresh-cut watermelon: postharvest physiology, technology, and opportunities for quality improvement

**DOI:** 10.3389/fgene.2025.1523240

**Published:** 2025-02-03

**Authors:** Ebenezer Quandoh, Karin Albornoz

**Affiliations:** Department of Food, Nutrition, and Packaging Sciences, Coastal Research and Education Center, Clemson University, Charleston, SC, United States

**Keywords:** *Citrullus lanatus*, fresh-cut processing, juice leakage, quality deterioration, cell wall, cell membrane

## Abstract

Watermelon (*Citrullus lanatus* L.) fruit is widely consumed for its sweetness, flavor, nutrition and health-promoting properties. It is commonly commercialized in fresh-cut format, satisfying consumer demand for freshness and convenience, but its shelf-life is limited. Despite the potential for growth in fresh-cut watermelon sales, the industry faces the challenge of maintaining quality attributes during storage. Fresh-cut processing induces a series of physiological and biochemical events that lead to alterations in sensory, nutritional and microbiological quality. A signal transduction cascade involving increases in respiration and ethylene production rates and elevated activities of cell wall and membrane-degrading enzymes compromise cellular and tissue integrity. These responses contribute to the development of quality defects like juice leakage, firmness loss and water-soaked appearance. They also drive the loss of bioactive compounds like lycopene, affecting flesh color and reducing nutritional value, ultimately culminating in consumer rejection, food losses and waste. Although great research progress has been achieved in the past decades, knowledge gaps about the physiological, biochemical and molecular bases of quality loss persist. This review article summarizes the advances in the study of physicochemical, microbiological, nutritional, and sensory changes linked to the deterioration of watermelon after processing and during storage. Different technological approaches for quality improvement and shelf-life extension are summarized: pre- and postharvest, physical, and chemical. We also discuss the advantages, disadvantages and challenges of these interventions and propose alternative directions for future research aiming to reduce qualitative and quantitative fresh-cut watermelon losses.

## 1 Introduction

Watermelon (*Citrullus lanatus* L.) is a widely cultivated fruit from the Cucurbitaceae family. It is one of the economically leading fruit crops globally, with a world production of 100 million tons, and China as the top producer (Faostat, 2022).

Watermelon consumption has increased in the last decade due to its juicy and fresh pulp, appealing color, high water content, and flavor in addition to its bioactive compounds such as lycopene and citrulline (Perkins-Veazie et al., 2012a; Petrou et al., 2013; Hong et al., 2015; Nelson et al., 2022). In the United States, watermelon is the most preferred melon type, accounting for over 67% of total melon availability, and it is the sixth most consumed fruit, purchased by 53% of households (Weber et al., 2023; Liu et al., 2024). However, its relatively large size limits portability and affects the purchasing decisions of customers (Hu et al., 2022). This has created an avenue for convenience, making fresh-cut watermelon a viable option. Fresh-cut or minimally processed produce are fruits and vegetables subjected to cutting, shredding, or peeling to provide convenient ready-to-eat or ready-to-cook servings (Watada and Qi, 1999; Brecht et al., 2003).

In the US, fresh-cut watermelon accounts for 14.7% of total watermelon sales, increasing by 18.3% in the 2023-2024 cycle (United Fresh Produce Association, 2020; National Watermelon Promotion Board, 2024). Despite the potential of the fresh-cut watermelon market to continue expanding, quality degradation during storage poses a challenge to the industry (Sipahi et al., 2013; Mendoza-Enano et al., 2019). Fresh-cut processing induces a dramatic metabolic remodeling that leads to changes in color, texture, and flavor and increases watermelon susceptibility to spoilage (Mao et al., 2006; Hu et al., 2022). These alterations compromise marketability and shorten shelf-life, contributing to sales stagnation in recent years (Artés-Hernández et al., 2021). Ultimately, quality loss leads to postharvest losses and waste (Wang et al., 2018; Hu et al., 2022; Wu et al., 2022).

The implementation of pre- and postharvest approaches applied directly to fresh-cut watermelon, or to whole fruit before processing has provided insights into the biological basis of postharvest quality loss and offered alternatives for shelf-life extension. Some of these strategies include grafting (Roberts et al., 2005), cutting tools and processing formats (Mcglynn et al., 2003; Petrou et al., 2013; Artés-Hernández et al., 2021; Lee, 2021), exogenous application of chemical agents (Kaveh, 2016; Ajami and Nemati, 2024; Jacuinde-Guzmán et al., 2024), modified atmosphere packaging (Cartaxo and Sargent, 1998; Lee, 2021), and irradiation treatments (Wang et al., 2018; Shi et al., 2020; Artés-Hernández et al., 2021; Jaiswal and Srivastava, 2024).

This literature review aims to consolidate the knowledge about the mechanisms underlying quality degradation in fresh-cut watermelon during storage and strategies for quality maintenance. We also highlight current research gaps, challenges, and avenues for future research.

## 2 Watermelon quality traits altered by fresh-cut processing

**FIGURE 1:**
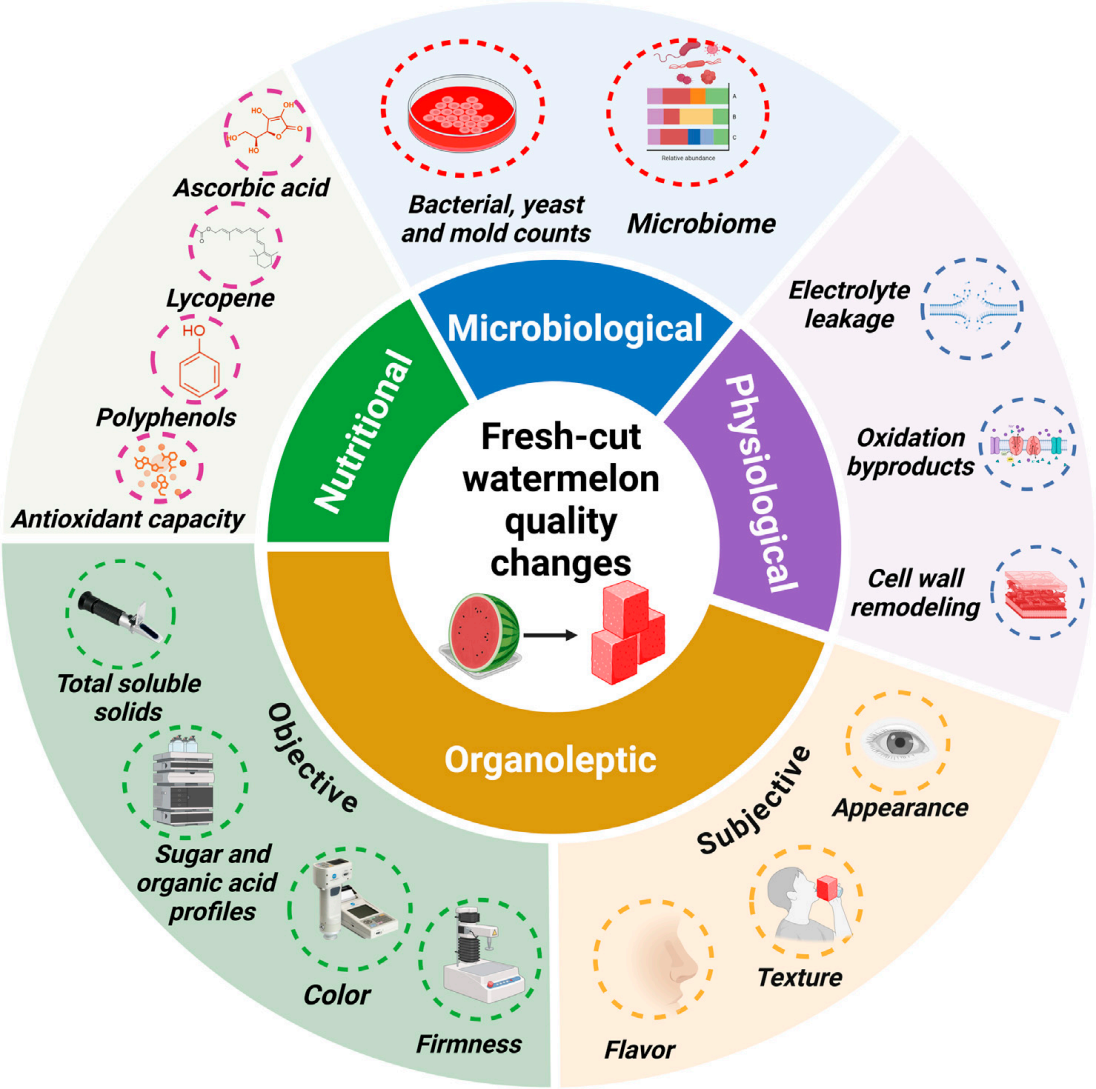
Organoleptic, nutritional, and microbiological quality parameters and physiological changes commonly reported in fresh-cut watermelon studies.

The quality of fresh-cut watermelon is underscored by a combination of attributes such as appearance, texture, and flavor, as well as nutritional value and food safety considerations that influence consumer perception. Fresh-cut operations promote physical and physiological changes (Figure 1) that lead to quality alterations and culminate in reduced shelf-life and marketability.

### 2.1 Juice leakage

**FIGURE 2:**
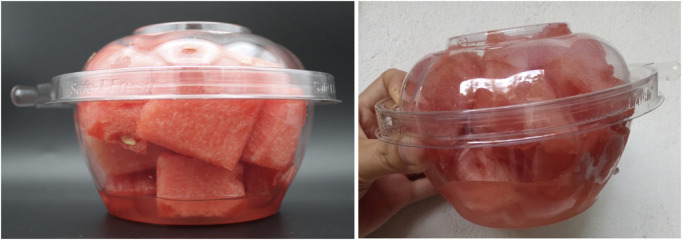
Juice leakage in fresh-cut watermelon. Juice accumulates at the bottom of containers during storage, increasing in volume and turbidity.

Also known as “drip loss” or “purge,” juice leakage is a common phenomenon during the storage of fresh-cut watermelon that affects its quality, safety, and marketability (Figure 2). Juice from the fruit cells migrates to and deposits at the bottom of containers by gravity, creating an undesirable appearance associated with freshness loss (Artés-Hernández et al., 2010; Wang et al., 2018). Juice leakage ranges an average of 4%–10% during storage on a fresh weight basis (Wang et al., 2018) and is influenced by ripening stage and storage temperature (Petrou et al., 2013). Perkins-Veazie and Collins, (2004) reported slightly higher values attributed to cultivar-dependent variations. Herein, during 10 days of storage at 2°C, fresh-cut samples from the seeded cultivar “Summer Flavor 800” presented 13% juice leakage in contrast to 11% observed in seedless “Sugar Shack” after 2 days of storage. No significant changes were observed beyond this point. In addition, leaked watermelon juice experiences changes throughout storage that act as an indicator of shelf-life. The juice contains sugars that create a favorable environment for microbial growth and anaerobic fermentation (Perkins-Veazie et al., 2012b). Increases in juice turbidity, i.e., the degree of clarity or cloudiness of the purge, correlate with higher microbial load, an indicator of food safety, as well as with declines in sensory quality and consumer preference (Gombas et al., 2017; Mohamad Salin et al., 2022). Despite the relevance of this quality defect in fresh-cut fruit, changes in the quality characteristics of leaked juice in watermelon have been scarcely examined in the literature.

### 2.2 Texture and firmness

Textural characteristics such as firmness, crispiness and juiciness are important indicators of the freshness and quality of fresh-cut produce including watermelon (Francis et al., 2012; Mendoza-Enano et al., 2019). Changes in texture are closely associated with alterations in cell wall structure and mechanical properties, particularly in fleshy fruits (Toivonen and Brummell, 2008). During ripening, pectin solubilization and depolymerization largely affect texture and quality (Vicente et al., 2007; Liu et al., 2023).

Extensive research on flesh firmness has been conducted in watermelon. In the last decades, consumer demand for firmer flesh has propelled breeding programs to integrate this trait into newer cultivars, departing from heirloom varieties that have a softer texture (Davis and King, 2007). During fruit development, cell wall remodeling largely determines watermelon quality and texture. Studies on the interplay between the hormones abscisic acid (ABA) and indole-3-acetic acid (IAA), along with cell wall-modifying enzymes have led to the identification of relevant regulators of fruit firmness (Sun et al., 2020; Anees et al., 2021; 2023).

**FIGURE 3:**
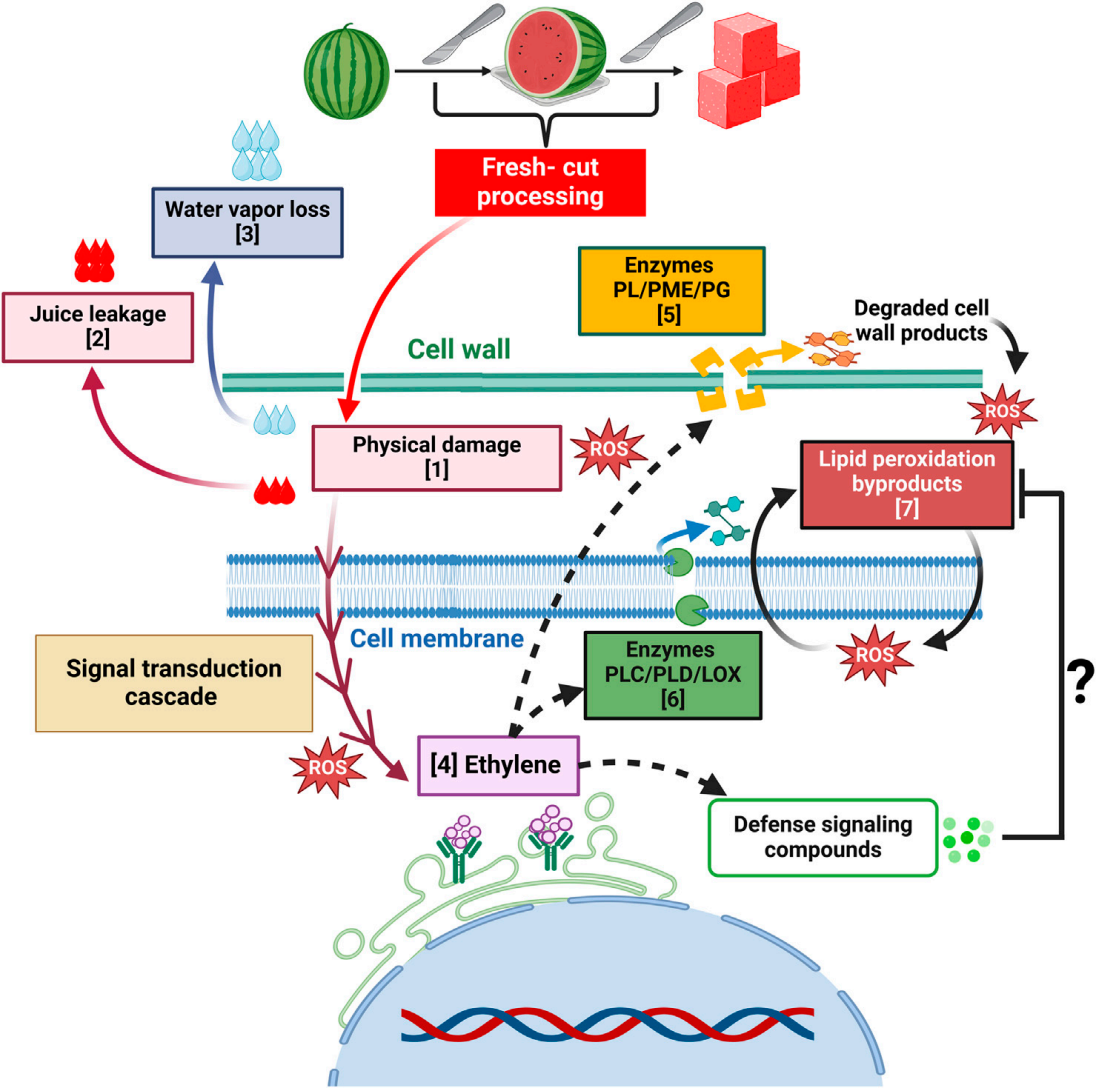
Texture-associated changes and proposed mechanisms influenced by fresh-cut processing. These alterations result from processes initiated by physical damage and tissue disruption [1] that promote juice leakage [2] and water loss in the form of vapor to the atmosphere [3]. Mechanical stress triggers a signal transduction cascade that stimulates physiological and metabolic responses, including increased respiration and ethylene production [4]. Enzymes like pectate lyase (PL), pectin methylesterase (PME), and polygalacturonase (PG) catalyze the degradation of the pectin fraction of the cell wall [5], generating various products. Other enzymes such as phospholipase C (PLC), D (PLD) and lipoxygenase (LOX) are involved in degrading cell membrane phospholipids [6] that result in the generation of lipid peroxidation byproducts [7] and reactive oxygen species (ROS) that participate in further peroxidation reactions. The dashed lines symbolize that further research is needed to determine if the events occur in parallel or are driven by ethylene. The question mark indicates that additional studies are required to determine if ethylene triggers defense-signaling compounds that detoxify or scavenge the byproducts of oxidative reactions, as seen in other fruits (Li et al., 2022; Wang et al., 2023).

In fresh-cut products, softening results from concerted processes initiated by physical damage and mediated by enzymes (Ragaert et al., 2007; Toivonen and Brummell, 2008) (Figure 3). Pectate lyase (PL), polygalacturonase (PG), and pectin methylesterase (PME) are the most widely studied and characterized pectinases that catalyze this disintegration (Karakurt and Huber, 2003; Toivonen and Brummell, 2008). Interestingly, in fresh-cut watermelon, cell wall dynamics have not been thoroughly described during storage, and only a few reports are available in the literature. Xisto et al. (2012) showed that PME activity did not vary in slices stored at 5°C for 10 days, and no PG activity was detected. Nonetheless, loss of firmness was observed along with an increase in soluble pectin, an indicator of cell wall disassembly. This observation suggests the action of alternative players in cell wall modification.

Loss of firmness due to decreased turgor is also reported in fresh-cut produce and linked to cell disruption, water loss and juice leakage (Hu and Jiang, 2007; Francis et al., 2012) (Figure 3). In watermelon, Myriah Mason et al. (2017) observed a slight, non-significant decrease in firmness in fresh-cut samples of four cultivars after a 5-day storage period at 5°C. In a study by Xisto et al. (2012), scanning electron micrographs of slices stored at 5°C revealed the release of liquid from the fruit tissue due to cell disintegration after 10 days. This observation aligned with an increased content of soluble pectin, juice leakage, and a greater rate of firmness loss.

Fresh-cut processing disrupts cell membranes, triggering physiochemical changes that compromise structural integrity (Figure 3). This leads to functional impairments, ultimately manifesting as altered texture (Brecht et al., 2003). In watermelon, these processes manifest as a loss of selective permeability, and greater ion or electrolyte leakage (Mao et al., 2004; Fan and Sokorai, 2005). Organelle decompartmentalization triggers the release of the enzymes phospholipase C (PLC) and D (PLD) and lipoxygenase (LOX), along with lipids that serve as substrates for further biochemical reactions (Brecht et al., 2003; Karakurt and Huber, 2004; Mao et al., 2004).

Malondialdehyde (MDA) is a byproduct of lipid peroxidation and a biomarker of oxidative stress and membrane damage (Brecht et al., 2003; Hu et al., 2021). Subsequent peroxidation of the resultant lipids leads to the formation of reactive oxygen species (ROS) that cause further membrane disruption and loss of cell integrity (Shewfelt and Rosario, 2000; Brecht et al., 2003) (Figure 3). Watermelon slices from five cultivars stored at 4°C for 5 days were assessed for membrane integrity via MDA, ROS content and peroxidase (POD) activity, which catalyzes the degradation of hydrogen peroxide (H_2_O_2_) to oxygen (O_2_) and water. Data showed that “Jingxin #1” and “8424 seeded” recorded the highest MDA contents. High POD activity, concomitant with low H_2_O_2_ and high O_2_ levels were shown by “Qilin” and the opposite in “8424 seeded”, thus highlighting a genotype-dependent response. Interestingly, cultivars that presented high MDA also recorded high juice leakage (Myriah Mason et al., 2017). Further research is needed to explore a potential correlation between these parameters.

### 2.3 Flesh color

Visual appearance is a pivotal criterion of consumer perception in the fresh-cut produce market, affecting purchase decisions and market value (Lund et al., 2000). The diversity of flesh colors of watermelon; red, pink, pale yellow, white and orange is underscored by carotenoid composition (Tadmor et al., 2005). In red-fleshed watermelons, α and β-carotene, phytofluene, phytoene, and lycopene are commonly reported carotenoids, with the latter being the most predominant (up to 97%) (Perkins-Veazie et al., 2001; Zhao et al., 2013; Ilahy et al., 2019).

Fresh-cut processing induces changes in watermelon flesh color that can be detected instrumentally or by sensory evaluations and consumer panels (Figure 1). Watermelon cubes monitored for 9 days of storage at 5°C were linked to low visual appearance scores by panelists and increased Hue values, dark red appearance and development of water-soaked areas compared to whole fruits (Gil et al., 2006). Perkins-Veazie and Collins (2004) recorded increased L* and decreased a* values in cubes stored at 2°C for 10 days, denoting loss of lightness and redness, respectively. Correspondingly, reductions in lycopene content were observed, however, the correlation with objective color descriptors was weak. In another study, in addition to a 6%–16% lycopene loss, fresh-cut cubes stored at 5°C for 11 days displayed low subjective sensory scores for appearance and overall quality due to off-color development and translucent damaged edges (Artés-Hernández et al., 2010). Nevertheless, cultivar-dependent variations in flesh color may present a limitation to objective color measurements. Evaluation of this trait requires complementary indicators to comprehensively assess fluctuations during storage.

### 2.4 Flavor

Flavor is the combined perception of taste and aroma and determines consumer acceptance (Ramirez et al., 2020). The primary determinants of fruit taste are the levels of sugar and organic acids and their balance, which influence sweetness and acidity (Figure 1). The aroma, on the other hand, is primarily determined by the quantity and diversity of volatile organic compounds (VOCs) (Clark, 1998; More et al., 2020). As respiratory substrates, these components are depleted due to the elevated respiration rates resulting from cutting operations, affecting the overall flavor of fresh-cut watermelon (Xisto et al., 2012; Hu et al., 2022).

Total soluble solids (TSS) is a commonly used estimator of the sweetness and sugar content of watermelon fruit (Huang et al., 2022). The produce industry is highly reliant on this parameter, and hence, its management is crucial (Tarazona-Díaz et al., 2011). In fresh-cut watermelon, TSS responses during storage are variable and influenced by genotype and handling conditions. During 10 days of storage of fresh-cut cubes at 2°C, Perkins-Veazie and Collins (2004) observed a decline in TSS after 2 and 7 days in “Sugar Shack” (seedless) and “Summer Flavor 800” (seeded), respectively. In contrast, Myriah Mason et al. (2017) reported that sliced samples from four out of five cultivars did not exhibit significant TSS changes, and one showed an increase after 5 days at 5°C. Gil et al. (2006) found that cubes and whole fruits stored at 5°C did not differ in TSS after 6 days. However, after 9 days, whole fruits had higher TSS than the cubes.

Studies on the aroma profile of watermelon have been primarily carried out on fruit immediately after cutting (Beaulieu and Lea, 2006; Liu et al., 2018; Fredes et al., 2024) and on juice (Xiaowei et al., 2012; Liu et al., 2018), with fewer reports on fluctuations throughout postharvest storage. Xisto et al. (2012) analyzed the volatile profile of fresh-cut samples during a 10-day storage period at 5°C using Gas Chromatography-Mass Spectrometry (GC-MS) from Solid-Phase Microextraction (SPME). Six-carbon and nine-carbon alcohols and aldehydes associated with the characteristic aroma of watermelon were detected during this period. Four of these compounds, (E,Z)-2,6-nonadienal, (Z,Z)-3,6-nonadienol, Z-6-nonenol and E-2-nonenal declined progressively, proportional to the decrease in aroma perceived by an untrained sensory panel.

### 2.5 Bioactive compounds

Bioactive compounds contained in watermelon, such as lycopene, vitamin C and polyphenols, have antioxidative, anti-inflammatory and antimicrobial activities associated with potential benefits for human health (Manivannan et al., 2020; Meghwar et al., 2024) (Figure 1). Fresh-cut processing has been shown to alter the levels of these compounds; however, there is little published data on fluctuations of other relevant phytonutrients in watermelon, like β-carotene and citrulline, during postharvest storage.

For lycopene, Perkins-Veazie and Collins, (2004) described a 6%–11% decrease in cubes stored at 2°C for 10 days. A similar trend has been reported in fresh-cut samples of “Tri-X 313” after 3 and 6 days (Gil et al., 2006), and “Crimson Sweet” after 14 days at 5°C (Kaveh, 2016).

Phenolic compounds, apart from their beneficial role to humans, also play a vital role as secondary metabolites in response to biotic and abiotic stress. Fresh-cut operations trigger phenolic alterations; loss, biosynthesis and metabolic turnover (Babic et al., 1993; Hodges and Toivonen, 2008). Decreases in total phenol content were observed in fresh-cut watermelon during storage at 5°C for 5 days (Gómez et al., 2017; Artés-Hernández et al., 2021) and 11 days (Artés-Hernández et al., 2010). Nonetheless, flesh discoloration is not commonly associated with enzymatic browning as this fruit has a relatively low content of phenolic compounds (Gil et al., 2006).

Ascorbic acid (AA) is a key biomarker of oxidation in fresh-cut fruits and vegetables (Barth et al., 1993). In fresh-cut watermelon, AA declined up to 6% after 11 days of storage at 5°C (Gil et al., 2006; Artés-Hernández et al., 2010).

### 2.6 Microbiological quality

Ensuring microbial safety is a major priority of the fresh-cut industry. The rising incidence of fresh-cut associated alimentary toxicosis outbreaks has become a growing public concern (Li et al., 2013; Hu et al., 2022). In the United States, a multistate outbreak of *Salmonella* on fresh-cut fruit salad resulted in 60 cases across six states in 2018 (Food and Drug Administration, 2018); therefore, ensuring quality and microbial safety is crucial for the industry. Tissue disruptions due to fresh-cut operations facilitate the release of cellular contents that migrate to adjacent intercellular spaces and non-wounded tissues (Saltveit, 2002; Brecht et al., 2003; Iturralde-García et al., 2022). This movement and accumulation provide a nutrient-rich environment for the growth of both spoilage and pathogenic microorganisms, culminating in reduced quality and shelf-life as well as public health risks (Brecht et al., 2003; Yousuf et al., 2020).

In watermelon, fresh-cut-induced changes in microbial populations have been described during refrigerated storage (Figure 1). Increased counts of yeast, mold and enterobacteria were reported in “Fashion” (Artés-Hernández et al., 2021), “Texiaofeng” and “8424 seeded” after 7 days of storage at 5°C (Myriah Mason et al., 2017). In “Fashion” cubes, the growth dynamics of different microbial classes, mesophilic and psychrophilic, showed an increase in population counts after 4–6 days, reaching the maximum by the end of the 12-day storage period at 5°C (Artés-Hernández et al., 2010).

The potential of sanitation, good manufacturing practices, temperature management as well as the application of physical and chemical treatments to address microbial and food safety risks during storage has been explored (Soliva-Fortuny and Martín-Belloso, 2003; Lee, 2021). These approaches are summarized in section 3.

## 3 Technologies and practices to extend the shelf-life of fresh-cut watermelon

Fresh-cut watermelon meets consumer demand for nutrition, sensory quality and convenience; however, its short shelf-life poses a challenge. Different strategies at the pre- and postharvest levels have broadened our understanding of the factors contributing to quality decline during storage. Some of these approaches have been directly evaluated on cut watermelon, while others have been applied to whole fruit prior to cutting, followed by processing and subsequent assessment of postharvest quality. They have been summarized in Table 1.

**TABLE 1:** Overview of outcomes of applied techniques to improve the quality and shelf-life of fresh-cut watermelon.

Type of strategy	Cultivar	Treatment	Storage conditions of fresh-cut fruit	Physiological and quality effects	References
Pre-Harvest					
Grafting	NP	Grafting watermelon onto four rootstocks	5°C for 10 d	Fresh-cut fruit from grafted plants showed better firmness retention	Roberts et al. (2005)
Postharvest					
Post-cut temperature	‘Honey seeded’	Cube storage at different temperatures	4 or 13°C for 6 d	Reduced *Listeria monocytogenes* growth at 4°C	Moreira et al. (2023)
	NP	Cube storage at different temperatures	5 or 25°C for 21 d	Reduced *Escherichia coli* growth at 5°C	Del Rosario and Beuchat (1995)
	NP	Cube storage at different temperatures	1 or 3°C for 10 d	Storage at 3°C resulted in 50% less juice leakage incidence and chilling injury	Sargent (1998)
	NP	Cube storage at different temperatures	1, 3, 7, 11, 15, or 30°C for 10 d	Cubes stored at 3°C maintained visual quality and flavor, reduced juice leakage and microbial load; shelf-life extended 3 d	Fonseca et al. (2004)
Post-cut hold	NP	Cube holding at 20°C for 2 h before refrigeration	4°C for 12 d	No effect on firmness and juice leakage. Reduced visual quality, aroma and marketability	Lee (2021)
	NP	Cube holding at 22°C for 3 or 5 h before refrigeration	5°C for 5 h	*Salmonella* growth was reduced	Ukuku and Sapers (2007)
Pre-cut hold	NP	Whole watermelons kept at 4, 20 or 30°C for 2 d before fresh-cut processing	4°C for 7 d	Cubes processed from whole fruits held at 20°C had higher aroma scores and lower juice leakage	Lee (2021)
Cutting size	NP	Cube sizes of 2, 2.5 and 3 cm	4°C for 7 d	Larger cubes sizes had the highest acceptable appearance and aroma, reduced respiration rate and better firmness retention	Lee (2021)
	‘Fashion’	Cube sizes of 1, 2, 4, and 8 cm	5°C for 7 d	Larger cubes size had reduced microbial counts and high levels of lycopene, total phenols and antioxidant capacity	Artés-Hernández et al. (2021)
Cutting tool	‘SugarTime’	Fresh-cut operation performed by water-jet or knife-cutting	4°C for 14 d	Water-jet reduced weight and firmness loss compared to knife-cutting No effect on color	Mcglynn et al. (2003)
Chemical	‘Sangria’	1000 ppm sodium hypochlorite dip of whole fruit for 1 min	4°C for 14 d	Reduction of microbial counts compared to control	Mcglynn et al. (2003)
	NP	Neutral pH electrolyzed water at 100 or 200 μL L^−1^ for 5 min	4°C for 7 d	200 μL L^−1^ reduced juice leakage, and maintained firmness and overall consumer acceptance	Lee (2021)
	NP	Fresh-cut samples exposed to H_2_ gas (4.2 and 42 μL L^−1^) for 4 h	4°C for 7 d	4.2 μL L^−1^ maintained consumer acceptance, reduced respiration rates, juice leakage and retained firmness relative to other treatments	Lee (2021)
	‘Millionaire’	Whole fruit exposed to 10 μL L^−1^ 1-MCP for 24 h before processing	10°C for 7 d	Reduced microbial growth, but no effect on respiration, electrolyte leakage, and firmness compared to untreated control	Mao et al. (2006)
	‘Millionaire’	Whole fruit exposed to 10 μL L^−1^ 1-MCP for 24 h followed by dipping of cut samples in 2% calcium chloride (CaCl_2_) for 5 min	10°C for 9 d	1-MCP + CaCl_2_ reduced respiration rate and maintained firmness but were less effective against electrolyte leakage compared to untreated control	Mao et al. (2006)
	‘Sugar Heart’	Whole fruits treated with 0.5 or 1 μL L^−1^ 1-MCP for 18 h + 10 μL L^−1^ ethylene for 5 d	5°C for 12 d	1-MCP inhibited ethylene-induced softening, reduced respiration rate, preserved visual quality, aroma volatiles and TSS relative to air-stored control	Saftner et al. (2007)
	‘Sugar Heart’	Whole fruit treated with 0.5 or 1 μL L^−1^ 1-MCP for 18 h + 10 μL L^−1^ ethylene for 5 d	5°C for 12 d	1-MCP inhibited ethylene-induced microbial growth and reduced juice leakage compared to air-stored control	Zhou et al. (2006)
	NP	Fresh-cut fruit dipped in calcium ascorbate (CaAsc) at 1, 5, 10 or 20% for 2 min	10°C for 8 d	1% CaAsc minimized weight loss, juice leakage and preserved flesh color	Lichanporn et al. (2014)
	NP	Fresh-cut fruit dipped or misted in 0.3 or 0.6 M CaAsc for 2 min	4°C for 10 d	0.3 M CaAsc maintained firmness and reduced juice leakage. Misting maintained consumer acceptance	Lee (2021)
	‘Fashion’	Cubes dipped in hot (45°C) or cold (5°C) 0.5% or 1% CaCl_2_ for 2 min	5°C for 8 d	1% CaCl_2_ hot-water dip inhibited microbial growth, maintained overall sensorial quality, reduced respiration and firmness loss	Aguayo et al. (2013)
	‘Sugar coat’	Cubes immersed in 100, 150, or 200 mg L^-1^ calcium as CaO and Ca(OH)_2_ nanoparticles (NP) for 3 min	5°C for 12 d	150 and 200 mg L ^-1^ Ca(OH)_2_-NP reduced water soaking, PG and PME activities, lipid peroxidation, enhanced polyphenol content, antioxidant capacity, and firmness retention	Jacuinde-Guzmán et al. (2024)
	NP	Cubes immersed in 1 or 2 mM salicylic acid for 2 min	4°C for 14 d	2 mM reduced weight loss, microbial growth and enhanced firmness retention	Ajami and Nemati (2024)
	NP	Cubes immersed in 0.5 or 1 mM citric acid for 2 min		1 mM maintained firmness, reduced weight light loss and microbial growth	Ajami and Nemati (2024)
	‘Crimson Sweet’	Cubes treated with 10% (v/v) saffron petal extract for 10 min	5°C for 14 d	Better retention of lycopene, visual quality and inhibited microbial growth compared to untreated control	Kaveh (2016)
	‘Sorento’	Slices treated with eugenol (250, 500, and 750 ppm) and carvacrol (100, 150 and 200 ppm)	15 or 25°C for 6 d	Eugenol and carvacrol applied at 750 and 200 ppm, respectively, inhibited growth of pathogenic fungi compared to other treatments	Šimović et al. (2014)
	NP	Slices dipped in clove basil leaf extract for 10 min	4°C for 9 d	Reduced microbial growth and polyphenol loss compared to untreated samples	Ebabhi et al. (2019)
	NP	Cubes treated with 0.0204, 0.0408 or 0.0612 g cinnamon oil (CO) or β-Cyclodextrin (CO-β-CD) per 100 g of watermelon	4°C for 4 d	0.0408 g CO-β-CD reduced weight loss, microbial growth, maintained flesh color, TSS and improved consumer acceptability compared to CO	Li et al. (2019)
	‘Royal Armada’	Cubes treated with 150–200 mg L^−1^ peracetic acid	3°C for 8 d	Microbial counts remained within safe limits, but TSS and subjective sensory scores decreased	Mendoza-Enano et al. (2019)
	NP	Fresh-cut sample treated with ozone at 4.2 mg dm^-3^ for 1, 2 or 3 min	4°C for 6 d	3 min exposure reduced microorganism growth, and reduced flesh color and vitamin C losses	Man and Huy (2008)
Edible coatings	NP	Cylinders coated with 0.5, 1.0 or 2g/100 g edible antimicrobial alginate-based edible coating	3°C for 15 d	2g/100 g sodium alginate reduced respiration rate and weight loss, maintained firmness, visual and organoleptic quality, but flavor was reduced	Song et al. (2023)
	NP	Cubes coated with agar/carrageenan-Nisin-based polysaccharide film	3°C for 18 d	Reduced microbial growth, firmness and vitamin C retention compared to uncoated samples	Sipahi et al. (2013)
Irradiation	‘Jingxin No. 3’	Cubes exposed to 10, 150 or 3000 Lux visible light	4°C for 5 d	3000 Lux reduced cell wall degradation, weight loss and drip loss. PG activity was inhibited but higher PL activity	Wang et al. (2018)
	‘Guomin No.2’	Cubes exposed to visible light: blue, yellow, green and red	4°C for 4 d	Red light reduced weight loss, water-soaking, maintained redness and firmness and delayed aroma loss. Visible light lowered antioxidant capacity compared to control (white light)	Shi et al. (2020)
	‘Abrusen’	Cubes exposed to 6 or 12 J cm^-2^ pulsed light	5°C for 15 d	12 J cm^-2^ reduced *E. coli* and *L. innocua* growth, and decreased ethylene production, but accelerated softness and loss of color relative to 6 J cm^-2^ and unexposed control	Ramos-Villarroel et al. (2012)
	‘Raspa,’ ‘Sangria’	Cubes exposed to 1.4, 4.1, 6.9 or 13.7 kJ m^−2^ UV-C light for 3min	3°C for 7 d	4.1 kJ m^−2^ reduced juice leakage and microbial population. Flesh color and organoleptic quality were maintained	Fonseca and Rushing (2006)
	‘Fashion’	Cubes exposed to UV-C at 1.6, 2.8, 4.8 or 7.2 kJ m^−2^	5°C for 12 d	1.6 and 2.8 kJ m^−2^ maintained organoleptic quality, lycopene and ascorbic acid and increased antioxidant capacity compared to other doses and unexposed control	Artés-Hernández et al. (2010)
	NP	Cubes exposed to 1.0 kGy electron beam dose	Ambient temperature or 5°C for 21 d	Flesh color, overall sensory organoleptic quality and firmness were better maintained; microbial growth reduced compared to unexposed control	Smith et al. (2017)
	‘Schrad’	Cubes exposed to 1 or 2.5 kGy gamma rays	5°C for 12 d	1 kGy had higher consumer acceptance and sweetness score	Landgraf et al. (2006)
	‘Mansfeld’	Cubes exposed to 0.5 or 1 kGy gamma radiation	4°C for 12 d	1 kGy lowered respiration and microbial growth, 0.5 kGy improved shelf-life by 4 d	Trigo et al. (2006)
	NP	Cubes exposed to gamma rays at 0.5, 1, 1.5, and 2 kGy	5°C for 8 d	1 kGy maintained visual quality, flavor, texture, and firmness relative to other treatments	Mohacsi-Farkas et al. (2006)
	NP	Disks exposed to 4, 6, 8, 10 or 12 J cm^−2^ pulsed light	N/A	4–6 J cm^−2^ presented higher firmness, phenol and antioxidant content compared to other treatments and control (unexposed); 10–12 J cm^−2^ reduced microbial growth and PME activity	Jaiswal and Srivastava (2024)
Irradiation + cutting size	‘Fashion’	Whole fruit exposed to UV-C at 1.6, 2.8 4.8 or 7.2 kJ m^−2^. Cutting sizes of 1, 2, 4 and 8 cm	5°C for 7 d	4.8 kJ m^−2^ and larger cut cylinders (≥4 cm) lowered respiration, juice leakage, microbial growth and improved acceptability. Lycopene, phenolic content, and total antioxidants were better retained	Artés-Hernández et al. (2021)
Chemical + irradiation	‘Millennium’	Fresh-cut pieces dipped in 2% malic acid for 2 min before 12 J cm^-2^ pulse light exposure	5°C for 15 d	Reduced microbial growth	Ramos-Villarroel et al. (2015)
MAP	NP	Cubes stored at 5% O_2_, 10% CO_2_, and 85% N_2_	4°C for 21 d	MAP retained flesh color, reduced bacterial, yeasts and mold growth, but lowered consumer acceptability relative to air storage	Smith et al. (2017)
	NP	Cubes stored at 7 kPa CO_2_ + 18 kPa O_2_ + 85 kPa N_2_	4°C for 9 d	MAP reduced respiration, water soaking, juice and ion leakage, and lipid peroxidation. Visual quality and aroma maintained, but no effect on TSS and firmness relative to air storage	Lee (2021)
	‘Royal Armada’	Cubes stored at 5% O_2_, 10% CO_2_, and 85% N_2_	3°C for 9 d	MAP reduced respiration but did not improve consumer acceptability	Mendoza-Enano et al. (2019)
CA	‘Millionaire’	Fresh-cut samples stored at 3% O_2_ + 5, 10, 15 or 20% CO_2_	3°C for 14 d	3% O_2_ + 15–20% CO_2_ reduced microbial growth and maintained firmness. 3% O_2_ + 5–10% CO_2_ lowered juice leakage and preserved color	Cartaxo and Sargent (1998)

Key: NP, not provided.

Fresh-cut or fresh-cut samples-means that the authors did not mention the cut shape.

UV: ultraviolet light.

MAP: modified atmosphere packaging.

CA: controlled atmosphere storage.

### 3.1 Pre-harvest approaches

Pre-harvest management practices such as irrigation (Zhou et al., 2023), fertilization (Aitbayeva et al., 2021), grafting (Yang et al., 2024), growing methods (Kyriacou et al., 2018), and others (Kahan, 2013; Zaaroor-Presman et al., 2020) have been widely reported to influence the postharvest physiology and quality of whole watermelon fruit. These strategies have been reviewed in detail by Kyriacou et al. (2018) and Nadeem et al. (2022). Interestingly, there is little information about the effect of pre-harvest practices on fresh-cut watermelon quality and shelf-life, and only a few reports are available in the literature. The effect of grafting, a technique where the root system from one plant, i.e., rootstock, is joined with the top part of another plant, i.e., the scion, was tested in fresh-cut watermelon during storage at 5°C for 10 days. Cubes from grafted cultivars presented greater firmness retention than those from the non-grafted plants. However, grafting had no effect on TSS, bacterial counts or lycopene content, which showed a decrease of up to 10% (Roberts et al., 2005).

### 3.2 Postharvest approaches

#### 3.2.1 Temperature management

Temperature is the most important factor influencing the postharvest quality of fresh produce (Kader, 2002). It exerts effects on metabolic and physiological processes leading to changes in texture, appearance, flavor, and microbial growth (Gidado et al., 2024). In fresh-cut watermelon, a storage temperature of 5°C or below is recommended to ensure microbiological quality (Fleming et al., 2005; Ukuku and Sapers, 2007). Cold chain breaks during distribution, retail and at the consumer level can expose the product to abusive temperature conditions that may jeopardize food safety. Similarly, lack of temperature management at processing sites, storage and sale points promotes the growth of various microorganisms, including human pathogens, increasing the risk of foodborne illness for consumers (Bai et al., 2024; Nketiah et al., 2024; Oluka et al., 2024). Del Rosario and Beuchat, (1995) investigated the microbial growth dynamics of inoculated *Escherichia coli* on cubes stored at 25 or 5°C for 21 days. Cubes stored at 5°C recorded lower *E. coli* counts compared to samples at 25°C. Likewise, reduced microbial growth of *Listeria monocytogenes* was achieved by storing cubes at 4°C compared to 13°C in a 6-day study (Moreira et al., 2023). Microbial inhibition of *Salmonella* has also been reported for cubed samples stored at 5°C in comparison to 10 or 22°C (Ukuku and Sapers, 2007).

Evidence also suggests that storage temperature drives changes in microbial populations and diversity. In this regard, findings from Feng et al. (2017) showed that in addition to inhibiting the growth of *E. coli* and *Salmonella*, natural microbiota was best maintained in cubes stored at 5°C, in contrast to 13°C and 25°C. Hu et al. (2022) monitored cubes on a 3-day study at 4 or 28°C using next-generation sequencing. Although microbial growth increased regardless of storage temperature, cubes at 4°C presented lower total bacterial counts than at 28°C. The microbiome analysis revealed that storage at 4°C had no effect on the initial microbial diversity, with proteobacteria and cyanobacteria as the dominant bacteria phylum. However, a distinct shift marked by elevated proteobacteria growth and emergence of firmicutes was observed in cubes at 28°C.

In terms of changes in organoleptic quality influenced by storage temperature, Sargent (1998) found that cubes refrigerated at 1°C presented 50% more juice leakage than those at 3°C for 10 days. In a different investigation, the shelf-life of cubes at 1, 3, 7, 11, 15, or 30°C was monitored for 10 days. Samples at 1 or 3°C had a longer shelf-life (10 d) than cubes at or above 7°C (<6 d), corroborated by less flesh darkening, off-odor development, juice leakage and microbial load (Fonseca et al., 2004). Mendoza-Enano et al. (2019) reported that panelists’ flavor liking scores of fresh-cut cubes decreased progressively during storage at 3°C and 7°C for 8 days. In parallel, objective measurements of odor-active volatiles revealed increased concentrations of compounds associated with reduced watermelon freshness, such as 2-butanone, 1-penten-3-one, hexanal and dimethyl trisulfide and acetophenone.

In an attempt to simulate commercial practices, the influence of pre- or post-cut hold temperature regimes prior to fresh-cut processing is further demonstrated in the following studies. Cut watermelon samples held at 20°C for 2 h before storage at 4°C were not commercially acceptable (visual quality and aroma) after 14 days compared to those that were not held. Nonetheless, the holding condition before storage had no effect on respiration and other quality traits like firmness and juice leakage (Lee, 2021). In another study, pre-cut storage of whole watermelons at 4, 20 or 30°C for 2 days led to quality alterations in cubes stored at 4°C for 7 days. Cubes from whole fruits incubated at 20°C presented better aroma and visual quality and lower juice leakage than the other treatments, while 4°C storage led to the highest firmness (Lee, 2021). Lee et al. (2018) incubated whole watermelons at 4, 21 or 30°C for 21 h, followed by fresh-cut processing and storage for 5 days at 4°C and 7°C. Findings revealed that across the pre-cut temperatures, 4°C-storage led to higher TSS than 7°C. In a study by Hu et al. (2022), TSS, glucose and fructose decreased more in cubes stored at 28 than at 4°C after 72 h. Furthermore, at 28°C, a concomitant decrease in pH was seen with the organic acids malate and citrate, while the fermentation markers, ethanol and lactate, increased. This trend contrasted with cubes stored at 4°C. In a report by Ukuku and Sapers (2007), holding cubes at 22°C for 3 or 5 h prior to storage at 5°C resulted in a progressive increase in *Salmonella* growth, whereas storing cubes immediately after processing inhibited its growth. Collectively, these reports highlight the relevance of refrigeration for organoleptic as well as microbiological quality maintenance.

#### 3.2.2 Cutting conditions and processing formats

Fresh-cut operations induce physical damage that alters the overall quality and shelf-life of produce (Portela and Cantwell, 2001; Incardona et al., 2024). In fresh-cut watermelon, different cut sizes have been shown to influence organoleptic quality during storage. Cylindrical-shaped fragments of 1, 2, 4, and 8 cm diameter stored at 5°C revealed that 1 and 8-cm samples had the lowest and highest visual appearance scores by panelists, respectively (Artés-Hernández et al., 2021). A similar finding by Lee, (2021) showed a differential effect of cut size (2, 2.5 and 3 cm) on cube quality during storage at 4°C. Here, the highest appearance and aroma scores were displayed by larger cubes (3 cm), and as the cut size increased, respiration was reduced, and firmness was better retained. The higher surface area to volume ratio of smaller fragments, as seen in melon, may explain some of these responses (Spadafora et al., 2019).

The presence of the rind, or the white layer surrounding the flesh, plays a significant role in the quality of fresh-cut watermelon. In this regard, Petrou et al. (2013) assessed the quality of slices with or without rinds stored at 4°C for 9 days. Findings revealed that while the presence of the rind had no effect on electrolyte leakage, slices with rind displayed 47% less juice leakage compared to rind-free slices. Furthermore, the presence of the rind minimized losses in TSS, total sugars, glucose, fructose and sucrose compared to rind-free slices.

Water-jet, a cold and high-pressure cutting technology is commonly used in the food industry for being safe and environmentally friendly (Henning, 1997). Mcglynn et al. (2003) studied the effect of this tool on the shelf-life and microbial quality of fresh-cut watermelon. Compared to knife-cut samples, water-jet led to firmness retention, but no differences were found in terms of flesh color and microbial counts during storage at 4°C for 12 days.

#### 3.2.3 Chemical treatments

The potential benefit of hydrogen gas (H_2_) in the maintenance of postharvest quality of fruits and vegetables has been reported via increased antioxidant capacity and reduced ethylene production (Hu et al., 2014; Gong et al., 2018). In fresh-cut watermelon, Lee et al. (2020) investigated the effect of an exogenous H_2_ application as a shock treatment (4 h after cutting) or under continuous application at 4.2 and 42 μL L^−1^ during cube storage at ∼5°C for 8 days. Regardless of the concentration, continuous H_2_ application was optimal for quality maintenance evidenced by better firmness retention, higher TSS and subjective quality scores. Respiration rates and water-soaking incidence were reduced in the continuous treatment compared with the shock treatment and control. Cubes treated with 4.2 μL L^−1^ H_2_ presented higher subjective aroma and appearance scores, less water-soaking incidence, and better firmness retention.

The inhibitor of ethylene perception 1-methylcyclopropane (1-MCP) plays an important role in maintaining the quality of fresh produce commercially. In a study by Saftner et al. (2007), whole watermelon was first treated with 1-MCP at 0.5 and 1 μL L^−1^, followed by ethylene treatment. Slices were obtained from the placenta, heart and rind and stored at 5°C for 12 days. Samples from all three parts treated with ethylene alone presented the lowest firmness and textural scores. Interestingly,1-MCP suppressed ethylene-induced softening in all tissues but did not influence firmness when no ethylene was applied. This was consistent with findings by Zhou et al. (2006), wherein 1-MCP exposure suppressed juice leakage and slowed microorganism growth in slices from ethylene-treated whole fruits. However, 1-MCP-associated benefits were not observed in samples obtained from whole fruits treated with 10 μL L^−1^ 1-MCP (without ethylene) prior to fresh-cut processing during 7 days of storage at 10°C (Mao et al., 2006).

The effect of potassium permanganate-based (KMnO_4_) ethylene removal sachets on the quality of fresh-cut watermelon was tested by Lee et al. (2020). KMnO_4_-treated cubes had higher crispness, aroma and appearance scores, and lower juice and electrolyte leakage than the air-stored (control) during storage at 4°C for 9 days.

In other conventional chemical treatments, calcium (Ca^2+^) and its salts are notable for their general role in postharvest quality maintenance, especially in decreasing produce softening by strengthening the cell wall (Glenn and Poovaiah, 1990; Braccini and Pérez, 2001; Soliva-Fortuny and Martín-Belloso, 2003; Pelloux et al., 2007). The application of different Ca-based products like salts and Ca-nanoparticles has been tried for microbial safety and shelf-life extension in fresh-cut watermelon with mixed results. The quality of cut samples dipped in 1, 5, 10 or 20% calcium ascorbate (CaAsc) solutions for 2 min showed a negative concentration-dependent relationship. Increasing CaAsc concentrations elevated weight loss, juice leakage and loss of redness (a* values) during 10 days of storage at 10°C. Nonetheless, 1% CaAsc was optimal to achieve a desirable effect on quality (Lichanporn et al., 2014). Findings by Lee et al. (2020) further corroborated this observation wherein cubes treated with 0.6 M CaAsc presented lower appearance and aroma scores, firmness and higher juice leakage than 0.3 M-treated cubes stored at 4°C for 10 days. In a study by Jacuinde-Guzmán et al. (2024), the quality and shelf-life of cubes immersed in 1% CaCl_2_ or Ca-based nanoparticles (CaO or Ca(OH)_2_-NP) were assessed during storage at 5°C for 12 days. Samples treated with 150 and 200 mg L^-1^ Ca(OH)_2_-NP exhibited lower water-soaking incidence and increased firmness by reducing PG and PME activities relative to other treatments and the control (water).

Evidence suggests that the benefits of calcium treatments are enhanced by hot-water dips. Dipping of watermelon cubes in hot (45°C) calcium chloride (CaCl_2_) at 1% displayed higher firmness retention, lower respiration, and reduced microbial growth during storage at 5°C for 8 days compared to cold-dipped cubes at 5°C (Aguayo et al., 2013).

The use of CaCl_2_ in combination with 1-MCP was examined during storage at 10°C for 9 days. Findings revealed that samples treated with 2% CaCl_2_ alone presented increased electrolyte leakage and respiration but these responses were suppressed by 1-MCP (Mao et al., 2006).

Salicylic acid (SA) is a phytohormone that has been shown to improve the postharvest quality and shelf-life of numerous horticultural crops when applied exogenously (Jing-Hua et al., 2008; Dokhanieh et al., 2013). Immersion of cubes in 2 mM SA effectively reduced weight loss and microbial growth and improved firmness retention by 82% compared to the untreated control during storage at 4°C for 14 days (Ajami and Nemati, 2024).

Citric acid is a naturally occurring organic acid used as an external treatment for fresh produce and is generally recognized as safe (GRAS). Watermelon cubes were immersed in 0.5 or 1 mM citric acid for 2 min and stored at 4°C for 14 days. A 3.24 log-fold reduction in microbial growth, lower weight loss and greater firmness retention were achieved in fruit treated with 1 mM citric acid (Ajami and Nemati, 2024).

Sanitizing whole watermelon in 1,000 ppm sodium hypochlorite for 1 min prior to cutting resulted in a 1.3-log reduction in colony forming units (CFU) of coliforms in fresh-cut pieces compared to the control (de-ionized water) during storage at 4°C for 7 days (Mcglynn et al., 2003).

Lee et al. (2020) evaluated the effect of chlorine-free sanitizers applied to whole watermelons on the quality of cubes. Samples treated with 200 μL L^−1^ neutral pH electrolyzed water (NEW) recorded lower juice leakage, and higher firmness and subjective sensory scores for aroma and appearance compared to those treated with water (control) after 10 days of storage at 4°C.

Peracetic acid at 150–200 mg L^−1^ was applied as a sanitation treatment to watermelon cubes. Samples were sealed with non-perforated film, stored at 3°C for 1 and 8 days, and compared to untreated cubes. Microbial counts remained within safe limits in both groups, but subjective sensory scores and TSS content were reduced in sanitized samples. Respiration rates were also higher in the sprayed group. These responses were attributed to the potential wash-off of flavor-related compounds induced by the post-cut sanitation step (Mendoza-Enano et al., 2019).

The effect of ozone on the microbial quality of cut watermelon was tested by Man and Huy (2008). Fresh-cut samples were ozone-treated at a concentration of 4.2 mg dm^-3^ (30°C, 1 atm) for 1, 2 or 3 min and stored at 4°C for 6 days. Findings revealed a time-dependent effect in microbial reduction within the treatments. Furthermore, samples treated for 3 min showed the highest reductions in aerobic bacteria (1-log), yeasts and mold counts (1.5-log), compared to the untreated control.

#### 3.2.4 Plant extracts and essential oils

Plant extracts are bioactive chemical-containing mixtures obtained through water and/or organic solvent extraction (Das et al., 2010). In postharvest management, their application has improved produce quality and shelf-life, making them a viable alternative to conventional chemical treatments (Das et al., 2010; El Khetabi et al., 2022).

Findings from Kaveh (2016) showed the antimicrobial and quality preservation potential of saffron petal extracts on fresh-cut watermelon stored at 5°C. Cubes treated with 10% (v/v) extracts for 10 min reduced lycopene degradation, preserved visual quality, and inhibited microbial growth. Similarly, cubes dipped in clove basil leaf extracts for 10 min prior to storage at 4°C had lower microbial growth and polyphenol losses compared to untreated samples (Ebabhi et al., 2019). In another study, eugenol and carvacrol essential oils applied at 750 and 200 ppm inhibited the growth of pathogenic fungi *Aspergillus carbonarius* and *Penicillium roqueforti* (Šimović et al., 2014). The application of cinnamon oil (CO) at 0.0204, 0.0408 or 0.0612 g per 100 g of watermelon or as an inclusion complex with β-Cyclodextrin (CO-β-CD) was investigated for quality preservation of cubes stored at 4°C for 4 days. Weight loss increased throughout storage; however, 0.0408 g CO-β-CD resulted in the least weight loss compared to the other treatments. Similarly, this concentration was more effective in preserving flesh color and TSS while limiting microbial growth, keeping total counts within acceptable limits (Li et al., 2019).

A drawback of essential oils application in foods is the potential trade-off in sensory quality. However, no changes in this regard were reported due to the low concentration (<1000 ppm) of eugenol and carvacrol used in fresh-cut watermelon studies (Šimović et al., 2014). In contrast, CO application resulted in low subjective flavor ratings. Interestingly, CO-β-CD improved flavor and consumer acceptance scores, likely due to its ability to absorb CO’s strong odor. (Li et al., 2019).

#### 3.2.5 Edible coatings

Edible coatings are thin, edible films of thickness less than 0.3 mm that are applied to produce surfaces to function as protective barriers. They are considered a type of active packaging because they provide a physical barrier to gas between the product and the environment. In addition, they can deliver additives and nutrients with biological activity that improve produce safety and quality (Han, 2014; Trajkovska Petkoska et al., 2021).

In fresh-cut watermelon, Song et al. (2023) evaluated the shelf-life and microbial quality of cubes coated with an agar/carrageenan (AG/CA) polysaccharide-based film or AG/CA loaded with the antimicrobial agent Nisin (AG/CA-N) during storage at 4 or 20°C for 18 and 6 days, respectively. Microbial analysis revealed that AG/CA-N effectively inhibited the growth of spoilage microorganisms *Staphylococcus aureus* and *L. monocytogenes*. Likewise, AG/CA-N outperformed AG/CA and the uncoated control in firmness and vitamin C retention. In a different study, the efficacy of a sodium alginate (0.5, 1.0 or 2 g 100 g⁻^1^)-based multilayered edible coating containing the antimicrobial agent trans-cinnamaldehyde was evaluated during 15 days of storage at 4°C. Findings revealed that the coating inhibited the growth of psychrotrophic microbes, yeasts and molds compared to the uncoated control, with the 2 g 100 g^-1^ concentration achieving the most significant reductions. A positive correlation between alginate concentration, firmness retention, respiration rate and overall acceptability was observed (Sipahi et al., 2013). Regarding the consumer acceptance of the tested coatings, the outcomes were heterogeneous. The 2 g 100 g^-1^ coating was the least preferred due to its appearance. Consistent with objective color measurements, the 0.5 and 1.0 g 100 g⁻^1^-coated samples ranked higher than the uncoated control. Similarly, the control and 1 g 100 g⁻^1^-coated samples received high flavor scores, whereas the 2 g 100 g^-1^ treatment had the lowest values explained by flavor changes imparted by trans-cinnamaldehyde. Altogether, these findings highlight the importance of material selection and dosage to achieving quality preservation in combination with meeting consumer sensory appeals.

#### 3.2.6 Irradiation treatments

Irradiation is a non-thermal food processing technology commonly used to achieve sterilization, modification and/or preservation of food qualities (Diehl, 2002). Based on the electromagnetic wavelength and frequency, this technology is classified as non-ionizing or ionizing irradiation (Lewandowski, 2001). Non-ionizing irradiation, such as visible light and ultraviolet (UV), primarily uses thermic effects produced by these rays. On the other hand, ionizing irradiation—electron beams and X-rays—acts via alterations in molecular structures, achieving microbial reduction and extended shelf-life (Zhong et al., 2021; Liu et al., 2023). These different methods have been applied to fresh-cut watermelon to assess their effects on quality and shelf-life.

##### 3.2.6.1 Visible light

Wang et al. (2018) exposed fresh-cut watermelon to visible light at 10, 150 (control), and 3000 Lux to simulate supermarket cabinet storage conditions for 5 days at 4°C. The 3,000 Lux treatment showed the lowest cell wall degradation ratio, PL and PG activities, electrolyte leakage, as well as fresh weight and drip loss among treatments. The exposure of cubes to blue, yellow, green and red light was evaluated during storage at 4°C for 4 days. Red light exposure led to significant firmness, color, appearance and aroma retention. Moreover, weight loss and water-soaking symptoms were reduced by 50% and 67%, respectively (Shi et al., 2020).

##### 3.2.6.2 Pulsed light

Pulsed light irradiation involves the application of very short to a high-intensity spectrum of light covering UV (100–400 nm), visible (400–700 nm), and infrared (700–1100 nm) for the preservation of produce shelf-life (Dunn et al., 1995; Elmnasser et al., 2007; Mahendran et al., 2019; Yao et al., 2023). In watermelon, pulsed light (12 J cm^-2^, 180-1,100 nm) applied to fresh-cut pieces resulted in a 3-fold log reduction of *E. coli and Listeria innocua* populations, decreased ethylene production and maintained firmness compared to the untreated control during 15 days of storage at 5°C (Ramos-Villarroel et al., 2012). Similarly, the use of pulsed light (12 J cm^−2^, 180-1,100 nm) in combination with 2% malic acid resulted in greater microbial reduction than their individual treatments and the untreated group by the end of 15 days of storage at 5°C (Ramos-Villarroel et al., 2015).

##### 3.2.6.3 Ultraviolet (UV) irradiation

UV is one of the most common irradiation preservation technologies in the fruit and vegetable industry (Liu et al., 2023). In the US, UV-C is widely used because it is environmentally friendly and toxic-residue-free (Rhim et al., 1999; Food and Drug Administration, 2002).

In watermelon, Fonseca and Rushing (2006) found that UV-C irradiation (4.1 kJ m^−2^) was more effective in inhibiting microbial growth than chlorine and ozone in cubes at the end of 8 days of storage at 3°C. UV-C also reduced juice leakage and loss of red flesh color. Artés-Hernández et al. (2010) assessed the overall quality, microbial, and bioactive compound alterations induced by UV-C exposure post-cutting during storage at 5°C for 12 days. A general reduction in bacterial counts was achieved by all treatments (1.6–7.2 kJ m^−2^) in a dose-dependent manner. Low doses (1.6 and 2.8 kJ m^−2^) presented higher organoleptic quality and bioactive compound retention than higher doses. Additionally, the combination of UV-C and cut size was investigated on cylinders during storage at 4°C for 7 days. Results revealed an interaction between these factors, where the combination of 4.8 kJ m^−2^ and large cylinders (≥4 cm) led to delayed microbial growth, and greater lycopene and phenolic content retention (Artés-Hernández et al., 2021).

##### 3.2.6.4 Electromagnetic radiation

This irradiation technology uses high-energy electrons generated from the electromagnetic field to achieve insecticidal and bacteriostatic activity effects on agricultural products (Nam et al., 2019; Liu et al., 2023).

In watermelon, cubes exposed to 1.0 kGy electron beam radiation maintained firmness and color, reduced microbial growth and had higher consumer acceptability scores than the untreated control during 21 days of storage at 4°C (Smith et al., 2017). In a different study, cubes irradiated with 0.5 and 1.0 kGy gamma presented no difference in firmness, flesh color and TSS during storage at 4°C for 12 days, but 1.0 kGy effectively reduced respiration rates, and microbial growth up to 2-log fold. On the other hand, irradiation at 0.5 kGy extended shelf-life by four additional days compared to 1 kGy by improving appearance, aroma and flavor (Trigo et al., 2006). Similarly, maintenance of shelf-life and microbiological quality has been observed in fresh-cut watermelon exposed to gamma radiation during storage at 5°C for 8 days (Mohacsi-Farkas et al., 2006) and 7°C for 12 days (Landgraf et al., 2006).

#### 3.2.7 Modified atmosphere packaging (MAP) and controlled atmosphere (CA) storage

Reduction of oxygen (O_2_) and increase of carbon dioxide (CO_2_) levels to 1%–5% and 3%–20%, respectively, have been reported as effective conditions for microbial inhibition and shelf-life maintenance of fresh produce (Kader and Watkins, 2000). MAP alters the initial gaseous composition inside of a package with no further control, whereas CA has a higher degree of control of gas levels (Lee et al., 1995; Farber et al., 2003; Wilson et al., 2019).

In fresh-cut watermelon, MAP storage with 3%–5% O_2_ and 10% CO_2_ has been recommended for preserving quality (Gorny, 2003). Smith et al. (2017) investigated the microbial counts and sensory and consumer acceptability of fresh-cut cubes placed in 5% O_2_, 10% CO_2_, and 85% Nitrogen (N_2_) MAP and stored at 4°C for 21 days. The proliferation of microorganisms was reduced, and firmness was maintained more effectively than air-stored (control) samples. Similarly, MAP conditions of 7 kPa CO_2_ + 18 kPa O_2_ + 85 kPa N_2_ reduced respiration, improved appearance and aroma and decreased juice and ion leakage in comparison to air-stored cubes at 5°C for 8 days (Lee, 2021). On the other hand, Mendoza-Enano et al. (2019) reported that regardless of the use of lidding films, MAP conditions of 5% O_2_, 10% CO_2_, and 85% N_2_ did not improve consumer acceptability scores of cut samples stored at 3°C after 6 days.

Cartaxo and Sargent, (1998) monitored fresh-cut watermelon samples stored under CA conditions. Cubes were placed into sealed jars connected to a humidified flow-through gas system at 3% O_2_ + 5, 10, 15 or 20% CO_2_ for 15 days at 3°C. The combination of 3% O_2_ and 10%–20% CO_2_ controlled microbial growth and maintained firmness, but increased juice leakage and induced a dark and dull water-soaked appearance compared to other treatments. These observations suggest the occurrence of physiological injury by either low O_2_, high CO_2_ or a combination of both.

## 4 The potential of omics technologies for extending fresh-cut watermelon shelf-life

The advent of omics technologies, i.e., genomics, transcriptomics, metabolomics and epigenomics has become a powerful tool for identifying targets for improving the quality and shelf-life of fresh produce (Habibi et al., 2024), including watermelon (Guo et al., 2015; Gao et al., 2020). While most of the current reports have focused on improving the sensory quality of whole fruit, research on the maintenance of quality traits during the postharvest storage of fresh-cut watermelon remains largely unexplored.

Texture is a complex trait that has significantly benefited from omics-based approaches. Gao et al. (2020) investigated the molecular mechanisms underlying texture during watermelon fruit development in two varieties, “PI186490” (white pulp, bitter, dense and hard pulp, green rind) and “Sanbai” (white and soft pulp, white rind). “PI186490” presented higher firmness and cellulose, hemicellulose and pectin contents than “Sanbai”. Comparative transcriptomic analysis revealed that among the 10 pectin esterase genes identified, *Cla004896, Cla014927, Cla015505 and Cla023049* were differentially expressed during development and upregulated in “PI186490” relative to “Sanbai”. A similar trend was observed for two differentially expressed PG genes (*Cla009218* and *Cla010077*) and one PL gene (*Cla002573*). Anees et al. (2021) carried out weighted genes co-expression network analysis (WGCNA) of a near isogeneic line with high average flesh firmness “HWF” and an inbred line with low average flesh firmness “203Z”. The study revealed that three main gene networks were correlated with water-soluble pectin, cellulose, hemicellulose, and protopectin, all pertinent to the structural integrity and firmness of watermelon fruit. A total of eight genes were identified as involved in cell wall biosynthesis and ethylene signaling. Linkage mapping and comparative transcriptome analysis identified key genes controlling center flesh firmness located on chromosomes 2 and 8 respectively: *Cla012507*, a MADS-box transcription factor and potential candidate gene regulating cell wall contents, and *ClERF1*, predicted to encode an ethylene-responsive transcription factor (Zhou et al., 2024). In another study, Genome-wide association studies (GWAS) and bulked segregant RNA-Seq analysis (BSR-Seq) identified an auxin-responsive gene (*Aux/IAA*) associated with flesh firmness on chromosome 6 (Anees et al., 2024).

Genome-wide comparative expression analysis of the carotenoid biosynthesis pathway has revealed gene regulatory networks associated with flesh color. Li et al. (2020) reported that *Cla018767, Cla018768, Cla018769, Cla018770,* and *Cla018771* were linked to red-flesh color development in watermelon via the lycopene biosynthesis pathway. The lycopene β-cyclase, *ClCYB* gene, was identified as a key regulator of red flesh color (Wang et al., 2019). Downregulation of *ClCYB* via an antisense construct in a pale yellow-fleshed line changed the color to red, whereas *ClCYB* overexpression in a red-fleshed line, induced an orange color development accompanied by diminished lycopene accumulation (Zhang et al., 2020). Similarly, quantitative trait loci (QTL) analysis has contributed evidence to the identification of genomic regions responsible for watermelon fruit color (Mashilo et al., 2023). In two Korean inbred watermelon lines with unique color and fruit-type characteristics, 15 QTLs associated with fruit quality-related traits were identified, two and four for lycopene content and flesh color, respectively. Fine-mapping identified 33 genes, including *Cla97C01G008760,* annotated as a phytoene synthase, a candidate gene that is crucial for the regulation of flesh color (Nie et al., 2023). Shahwar et al. (2024) used GWAS and genotyping-by-sequencing (GBS)-based QTL analysis on 130 watermelon recombinant inbred lines. The study identified a major QTL on chromosome 4, *qFC-4.1*, as well as *Cla97C04G070940* (lycopene β-cyclase, *LCYB*) and *Cla97C02G039880* (pentatricopeptide repeat, *PPR*) as candidate genes for flesh color.

Flavor is one of the main traits determining watermelon consumption and a relevant target of breeding programs (Mashilo et al., 2022; Liu et al., 2024). Comparative transcriptome analysis revealed that the genes *Cla97C01G013600, Cla97C05G089700, Cla97C01G001290, Cla97C05G095170,* and *Cla97C06G118330,* involved in alcohol and aldehyde (ADH) biosynthesis, enhanced flavor and aroma formation of ripe watermelon fruit (Gong et al., 2021). In a study by Peng et al. (2025), differentially expressed genes involved in sugar and organic acid metabolic pathways were identified in four commercial watermelon genotypes, including trehalose-phosphate synthase/phosphatase genes *Cla97C11G223240* (*TPS1*) and *Cla97C07G136350* (*TPPJ*), sucrose synthase genes *Cla97C10G194010* (*SUS2*) and *Cla97C02G041890*.

Fresh-cut processing promotes unique physiological changes in watermelon that significantly alter its storability compared to intact fruit. This presents an opportunity for omics research to investigate the dynamics of quality-related traits during postharvest storage.

## 5 Research gaps and future perspectives

This article reviewed relevant studies on quality changes, technological interventions and emerging approaches to extend the shelf-life of fresh-cut watermelon. However, further research is imperative to gain a more comprehensive understanding of the biological mechanisms that determine quality loss. This knowledge is crucial for developing effective strategies to reduce postharvest losses and waste.

Research efforts to understand the mechanisms underlying fresh-cut watermelon quality decline have increased in the past two decades. Early studies primarily focused on characterizing the changes in sensory attributes such as color, flavor, and texture during postharvest storage (Fonseca et al., 1999; Perkins-Veazie and Collins, 2004; Gil et al., 2006). In the last 10 years, there has been a shift towards investigating the use of exogenous treatments to delay quality loss (Sipahi et al., 2013; Šimović et al., 2014; Ebabhi et al., 2019; Song et al., 2023; Jacuinde-Guzmán et al., 2024) and a greater focus on the role of aroma volatiles in consumer acceptance (Xisto et al., 2012; Mendoza-Enano et al., 2019). Nevertheless, only a few reports have examined the biological mechanisms involved in texture-related defects like juice leakage, beyond the influence of the hormone ethylene (Petrou et al., 2013) (Figure 3). Given the importance of texture on the sensory experience of fresh-cut produce (Barrett et al., 2010) and the role of juice leakage in consumer rejection of fresh-cut watermelon (Mao et al., 2006; Mphahlele et al., 2020; Lee, 2021), further studies in this direction are needed.

From a nutritional perspective, there has been a strong focus on investigating the changes in total polyphenols, ascorbic acid and lycopene content during postharvest storage (Perkins-Veazie and Collins, 2004; Gil et al., 2006), but other bioactive compounds have received considerably less attention. Watermelon represents a valuable source of citrulline, a non-essential, non-proteinogenic amino acid, as well as Vitamin A, flavonoids and β-carotene (Sorokina et al., 2021). Rising consumer awareness of the nutritional content of fresh produce represents an opportunity to explore approaches to improve the retention of these phytonutrients during postharvest storage in fresh-cut watermelon.

Numerous strategies to improve fresh-cut watermelon quality have been documented in the literature (Table 1). Pre-harvest and post-harvest practices and physical and chemical treatments applied before or after processing present advantages but also limitations that must be addressed to develop long-term solutions to quality loss. Most of the chemically-based treatments have been tested on the cut ready-to-eat fruit which raises legitimate concerns about regulations on dosage and concentration, potential toxicity and public health risks. Alternatively, exploring the use of elicitation approaches applied to whole watermelon fruit before processing could expand the range of available options to address quality loss. Elicitors are stress factors that can be applied in a controlled manner to the plant during cultivation, i.e., preharvest, or postharvest. They trigger stress responses by activating the synthesis and accumulation of secondary metabolites responsible for protecting cells against damage (Baenas et al., 2014). Exposure of whole fruits to elicitors has been previously tested in melon and resulted in quality maintenance during storage (Atress and Attia, 2011; Liu et al., 2022).

Many exogenous treatments have been tested at the laboratory scale, providing valuable insights into the quality traits affected by fresh-cut processing and physical damage (Table 1). However, translational research is imperative for enhancing industry competitiveness by converting these findings into practical applications. Analysis of the sustainability and environmental impact of the proposed technologies are commonly overlooked, and knowledge gaps persist. The scarcity of available cost reports presents an opportunity to conduct research that could inform on the economic feasibility of implementing these methods. As pointed out by Lee (2021), the processing industry is receptive to incorporating technologies that involve existing equipment and infrastructure. In this context, the storage of watermelon fruit under optimal temperature conditions—pre- and post-cut—still represents the most viable intervention to maintain the quality of the fresh-cut product.

Technological interventions to extend fresh-cut watermelon shelf-life often have trade-offs, as some traits are improved while others are impaired. Additional research involving sensory evaluations could optimize the development of solutions to quality loss that are aligned with market and consumer demands.

Gene editing through CRISPR-Cas9 could further expand our understanding of complex traits like firmness and flavor (Shipman et al., 2021), which are greatly compromised by fresh-cut processing. (Huang et al., 2011; Liu et al., 2016). Research efforts have advanced in this area by exploring methods that increase both transformation and editing efficiencies (Tian et al., 2017; Pan et al., 2022; Wang et al., 2024). Nevertheless, gene editing approaches specifically oriented to improve fresh-cut watermelon quality and postharvest traits are still in the early stages of development. Leveraging this tool in conjunction with omics technologies holds significant potential to develop robust, long-term solutions for mitigating quality loss during storage and extending shelf-life.

A multidisciplinary approach, integrating applied, molecular, and biotechnological resources, can provide a robust understanding of the regulatory mechanisms underlying fresh-cut quality decline, which can then be effectively integrated into breeding programs. Enhanced quality will not only expand market access, stimulate consumption, and drive sales growth but also contribute to food losses and waste reduction.

## 6 Conclusion

Consumer interest in fresh-cut watermelon, as an alternative to whole fruit is increasing as it meets demand for convenience. However, fresh-cut operations induce stress and trigger biological processes that ultimately lead to quality deterioration. Color loss, bioactive compounds decline, and textural alterations underscore organoleptic and nutritional quality traits impacted by processing. The literature has reported a strong influence of the genotype on the differences in the rate and magnitude of quality decline for most traits. To address the challenges imposed by quality deterioration in fresh-cut watermelon, different approaches for shelf-life extension have been tested in the last decades. Pre-harvest interventions, like grafting on different rootstocks improved or maintained the firmness of the resulting fresh-cut product. Postharvest techniques applied pre- or post-cutting, such as irradiation or chemical treatments, had heterogeneous effects in maintaining quality and ensuring microbiological safety when used alone or in combination. Low-temperature storage improved organoleptic quality retention during storage, consistently reducing microbial growth. The application of edible coatings and controlled and modified atmosphere packaging were effective in maintaining some quality traits but led to trade-offs in other aspects. Overall, treatment effectiveness has been disparate, thus highlighting the need for additional research efforts on alternative methods for quality retention that are cost-effective. The increased application of gene editing and omics technologies to watermelon represents a promising opportunity to shorten knowledge gaps on the molecular basis of quality loss, and to develop improved cultivars for the fresh-cut market that complement current breeding efforts.
